# Evaluation of prognostic significance of hematological profiles after the intensive phase treatment in pulmonary tuberculosis patients from Romania

**DOI:** 10.1371/journal.pone.0249301

**Published:** 2021-04-01

**Authors:** Simona Ştefanescu, Relu Cocoş, Adina Turcu-Stiolica, Beatrice Mahler, Andreea-Daniela Meca, Ana Maria Cristina Giura, Maria Bogdan, Elena-Silvia Shelby, Georgeta Zamfirescu, Catalina-Gabriela Pisoschi

**Affiliations:** 1 Clinical Analysis Laboratory, Clinical Emergency County Hospital Craiova, Craiova, Romania; 2 Department of Medical Genetics, University of Medicine and Pharmacy “Carol Davila”, Bucharest, Romania; 3 Institute of Pneumophtisiology “Marius Nasta”, Bucharest, Romania; 4 Department of Pharmacoeconomics, University of Medicine and Pharmacy of Craiova, Craiova, Romania; 5 Pneumology Department (II), University of Medicine and Pharmacy “Carol Davila”, Bucharest, Romania; 6 Department of Pharmacology, University of Medicine and Pharmacy of Craiova, Craiova, Romania; 7 Department of Preventive Dentistry, University of Medicine and Pharmacy “Carol Davila”, Bucharest, Romania; 8 Scientific Research Nucleus, Dr. Nicolae Robanescu National Clinical Centre for Children’s Neurorecovery, Bucharest, Romania; 9 Clinical Analysis Laboratory, Leamna Pneumophtisiology Hospital, Craiova, Romania; 10 Department of Biochemistry, University of Medicine and Pharmacy of Craiova, Craiova, Romania; The University of Georgia, UNITED STATES

## Abstract

We evaluated in this cohort study the predictive ability of 23 peripheral blood parameters and ratios for treatment outcomes after the 2-month intensive phase in patients with PTB. In 63 patients out of 90 that turned culture negative, a significant decrease in white blood cell count, neutrophils, monocyte, hemoglobin, platelet, plateletcrit, erythrocyte sedimentation rate, MLR, NLR, PLR and SII values after anti-TB therapy compared to pretreatment was observed (p <0.001). Logistic regression analysis generated a model of predictors consisting of nine covariates. Spearman’s correlation analysis revealed significant positive correlations between NLR with NEU (r = 0.79, p<0.01), SII with NEU (r = 0.846, p<0.01), PLT with SII (r = 0.831, p<0.01), PLT with PCT (r = 0.71, p<0.01) and MPV with P-LCR (r = 0,897, p<0.01) in 63 patients out of 90 that turned culture negative after 2 months of treatment. ROC curve analysis indicated that all areas under the curve (AUC) revealed no statistically significant results, except lymphocyte for culture conversion. In summary, here we observed a set of hematological parameters that declined significantly as the disease was treated in patients that turned culture negative. Despite some limitations, our findings are useful for further studies aiming to identify hematological profiles that could predict the treatment outcome.

## Introduction

Tuberculosis (TB), a chronic disease caused by Mycobacterium tuberculosis (Mtb), is the most common infectious disease today. Roughly 10.0 million new cases occurred worldwide in 2018 and the annual death rate is 1.6 million [1, 2]. TB is also one of the top causes of death, ranking it above HIV/AIDS [3]. Furthermore, it is estimated that about 2.5 billion people are living with latent TB (LBTI) [4, 5].

Tuberculosis has been considered a major public health burden over the past three decades with a slight global year-over-year decline by only 2%, despite the global implementation of directly observed therapy short-course (DOTS) [6–8].

Newly diagnosed TB cases across the globe are treated, according to the latest World Health Organization (WHO) guidelines, with a standard first-line treatment regimen (2HRZE/4HR) of four antibiotics, isoniazid (H), rifampicin (R), pyrazinamide (Z), and ethambutol (E)—consisting of a 2-month initial phase of (2RHZE), followed by a 4-month continuation phase (4RH) [9, 10].

The bacteriological status, sputum smear microscopy (SSM) conversion and/or sputum culture conversion (SCC), at 2 months is commonly used as a microbiological milestone to monitor the outcomes of tuberculosis treatment during the intensive phase and every 2 months during the continuation phase [11–14]. Non-conversion in these two biomarkers of the prognosis of treatment was associated with an unfavorable outcome, higher rates of treatment failure, including drug-resistance and relapse [15, 16]. Although, the conversion to negative sputum culture after 2 months of anti-TB treatment presents a sensitivity of only 40% and SSM has low sensitivity and low specificity to detect failure, there is no other TB biomarker that meets this level of qualification [17, 18].

Mtb infection is characterized by a cellular response that includes various manifestations reflecting the interplay between Mycobacterium tuberculosis bacilli (MTB) and the main effector cells of the host cellular immune defense mechanisms [19–21]. The host immune response to infection is thought to play a critical role in pathophysiology of TB resulting in a great variety of immunopathology, ranging from asymptomatic infection to disseminated disease and eventually patient death [22]. Systemic inflammation was observed in both pulmonary tuberculosis (PTB) and extrapulmonary tuberculosis (EPTB) and is characterized by increased concentrations in a variety of inflammatory markers in peripheral blood and a spectrum of proinflammatory cytokines, as well as chemokines [23].

In recent years, accumulating evidence has drawn attention to various blood inflammation-related parameters, which can be easily obtained by routine blood cell count (CBC) in order to be used in combination to predict cancer [24], TB [25, 26] or cardiovascular treatment outcomes [27]. The white blood cell (WBC) count, platelets and various relative ratios of different white cells, such as neutrophil/lymphocyte ratio (NLR), platelet/lymphocyte ratio (PLR) and monocyte/lymphocyte ratio (MLR), have been widely investigated in chronic inflammatory diseases including TB [28, 29]. For example, increased MLR in peripheral blood has been found to differentiate patients with active and latent TB from healthy controls [30] and has been reported as a marker of TB development in infants, adults, and women with HIV infection [29]. The NLR ratio has been reported to help distinguish between pulmonary TB and bacterial community-acquired pneumonia [31] and predict the risk of TB among HIV-infected persons [32].

Recent reports have assessed a new inflammatory index, the systemic immune-inflammation index (SII), that integrates peripheral lymphocyte, neutrophil and platelet counts into one index defined as neutrophil x platelet/lymphocyte, which can easily be evaluated from blood samples to better reflect the interplay between the local immune response and immune status [33]. In addition, other hematological parameters have been investigated in chronic inflammatory diseases such as mean corpuscular volume (MCV), red cell distribution width (RDW), mean corpuscular hemoglobin (MCH), mean corpuscular hemoglobin concentration (MCHC), MPV, platelet distribution width (PDW) and erythrocyte sedimentation rate (ESR) have been investigated in chronic inflammatory disease [34, 35]. The role played by components of the blood cell count such as red blood cells (RBCs) in inflammatory condition was under-evaluated. However, the use of these combined hematological markers that can reflect the systematic inflammatory response prior to the initiation of treatment and after the 2-month intensive phase in PTB patients has not yet been fully investigated.

Identifying the optimal combination of hematological markers that could be predictive for the response to treatment in PTB patients is paramount when evaluating the effectiveness of anti TB drugs. Therefore, the aim of this work was to examine the potential role of 23 complete blood cell count parameters in order to predict treatment outcomes after the 2-month initial phase in patients with PTB.

Here, we evaluate the predictive ability of a large panel of routine blood cell count parameters and indices for the TB treatment outcome. In addition, no other study used this combined set of blood cell count parameters to define a pattern that adequately correlates with the effectiveness of the drug treatment after the 2-month intensive phase. To the best of our knowledge, the present study is the first to evaluate the optimal cut-off values for some of these parameters in order to predict the treatment outcomes in patients with pulmonary tuberculosis.

## Materials and methods

### Study subjects

Between January 2019 and April 2020, we recruited for a cohort study all adult newly diagnosed PTB patients with smear microscopy and Mtb sputum culture who were admitted to two medical centers in Dolj County, Leamna Pneumophtisiology Hospital (Centre 1) and “Dr. Victor Babes” Infectious Disease and Pneumophtisiology Clinical Hospital (Center 2), Craiova.

The entire protocol was approved by the Ethics Committee of University of Medicine and Pharmacy of Craiova, affiliated to Dr. Victor Babes” Infectious Disease and Pneumophtisiology Clinical Hospital, Craiova, and in compliance with the Declaration of Helsinki and its amendments. Written informed consent was obtained from all study participants based on the approved protocol.

All patients underwent a chest Computer Tomography (CT) scan or a chest X-ray. The newly PTB patients were diagnosed based on the clinical manifestation and radiological features in accordance with national guidelines.

Data on age, gender, education and occupational status, alcohol consumption, tobacco use, TB symptoms or physical examination were recorded. **S1 Table** presents the socio-demographic characteristics of the PTB patients. Given the highest incidence of TB in our region in Romania we can consider our group of patients representative of a larger population.

The major exclusion criteria were as follows: 1) patients with extrapulmonary tuberculosis, 2) age under 18 years, 3) previous history of anti-TB chemotherapy, 4) death during anti-TB treatment, 5) patients with infectious, chronic inflammatory, hematological or autoimmune diseases or malignant pathologies, patients with liver or kidney disease, diabetes mellitus or other co-morbid diseases that affect hematological parameters, 6) HIV-positive patients, 7) patients treated with steroid or other immunosuppressive drugs at the time of their first admission 8) patients who interrupted the treatment or changed the treatment regime due to side effects and 9) those patients whose data was missing. To narrow our focus on a more accurate impact of the tuberculosis treatment we excluded the patients with various comorbidities because the various medications taken by these patients may alter the hematological parameters and because the comorbidities were associated with significantly lower successful treatment outcomes.

Finally, after considering all exclusion criteria, of 106 newly diagnosed PTB patients, 90 (N = 49, Center 1 and N = 41, Center 2) were enrolled and available for the treatment outcome analysis in our cohort observational study after the 2-month intensive phase based on the evaluation of the hematological profiles, **S1 File**.

### Microbiological assessment

Sputum samples were collected for acid-fast bacilli (AFB) smears and mycobacterium cultures at the time of enrollment (T0) and after 2 months of treatment (T2). AFB smear was undertaken using auramine–rhodamine staining and confirmed by Ziehl–Neelsen staining. Sputum culture was performed on solid and liquid medium. Sputum smear microscopy and sputum culture were recorded for all patients after the second month of treatment.

### Blood count measurement

Venous blood was drawn from all 90 patients before initiation of anti-TB therapy and after the intensive phase. Five milliliters of blood were collected in EDTA standardized tubes at these two time points. The hematological measurements were performed using Abacus 5 Analyzer with 5-part diff (Diatron, Germany). Hematological quality control materials were analyzed to ensure the quality of data. The laboratory uses a quality procedure consisting of twice-daily high, normal and low internal quality control (IQC). As a part of the quality management system, external quality controls (EQS) and an annual quality assurance are used. The laboratory is accredited by the Romanian National Accreditation System (Romanian Accreditation Association—RENAR) in accordance with international standard ISO 15189/2013.

The following parameters (**S1 File**) were recorded: 1) Red blood cell (RBC) count, hematocrit (HCT), Hb (hemoglobin), RBC indices [mean corpuscular volume (MCV), red cell distribution width, coefficient variation (RDW-CV), red cell distribution width, standard deviation (RDW-SD), mean corpuscular Hb (MCH) and mean corpuscular Hb concentration (MCHC)]; 2) Total white blood cell (WBC), lymphocytes (LYM), neutrophil (NEU) and monocyte (MON); 3) Platelets (PLT) count, plateletcrit (PCT), platelet distribution width, coefficient variation (PDW-CV) and platelet distribution width, standard deviation (PDW-SD) and platelet large cell ratio (P-LCR).

The neutrophil-to-lymphocyte (NLR), platelet-to-lymphocyte (PLR), monocyte-to-lymphocyte (MLR) ratios and systemic immune-inflammation index (SII) were calculated based on the absolute counts of lymphocytes (NEU; ×109/L), monocytes (MON; ×109/L) and platelets (PLT; x109/L) using the following equations: NLR = NEU/LYM, MLR = MON/LYM, PLR = PLT/LYM and SII = PLT x NEU/LYM [36].

Additionally, measurement of erythrocyte sedimentation rate ESR (Westergren method) was performed for all patients.

### Treatment

Each newly PTB diagnosed patient was treated using the same national 6-month regimen that is in line with WHO guidelines [10], consisting of the 2-month regimen (2HRZE) followed by the 4 the month regimen (4HR). Other specialized health professionals than the researchers involved in this study treated the patients. The researchers collected the data using specific questionnaires and obtained the informed consent from the patients for this study. No patient was lost to follow up during our study as we stated in the exclusion criteria. The majority of patients included in our study were lost to follow-up succeeding their discharge in communities following the 2-month regimen.

Following the intensive phase, the TB treatment outcome data is difficult to collect and incomplete considering the voluntary reporting. Moreover, the treatment has been self-administered in the continuation phase, when patients were found to have interrupted the therapy, by choice or for any other reason.

### Statistical analysis

Statistical analyses were performed with SPSS version 25.0 (SPSS Inc, Chicago, IL) and R (version 4.0.3).

Data was represented as mean (standard deviation) or median (interquartile range), when reported for continuous variables, and the number of subjects (n) and percentages (%), when reported for categorical variables. Checking for normality was done using Kolmogorov-Smirnov (K-S) test.

In accordance with the result of these tests, comparison among groups was analyzed with the Mann–Whitney U test (without normal distribution) and Paired-Samples T-test (with normal distribution). Categorical variables were compared using chi-square test. Two-sided P value of less than 0.05 was considered as statistical significance.

The relationship between variables was established using Spearman correlation. The Spearman correlation coefficients between the hematological parameters were calculated and visualized as a heatmap with hierarchical clustering to look for patterns. Correlogram with hierarchical clustering was created to display the strength and direction of all biomarker correlations. The R *corrplot* package was used to plot the correlogram, where correlation coefficients are colored according to the value: positive correlations were displayed in blue and negative correlations in red color [37]. Color intensity and the size of the circle are proportional to the correlation coefficients. We combined correlogram with hierarchical clustering to group closely correlated biomarkers.

Binary logistic regression analyses were performed to assess the independent predictors. All potential confounding factors were included in the logistic regression analysis using forward selection with the step-wise selection in SPSS. We started off with no predictors in the model and we tried them one at a time, starting with one with the lowest p-value, and then kept adding variables until none of the remaining ones were significant. We assessed whether the hematological parameters that reached statistical significance could delineate an association with the culture status using the logistic regression analysis. The logistic regression has highlighted the main variables as predictors of CULTURE after 2 months of TB treatment. Our dependent variable is culture and the occurrence of culture is dichotomous (positive or negative). Statistically, the logistic regression gives us the probability of TB patients providing a negative culture after 2 months of treatment. A value close to 0 means that culture is very unlikely to be negative, and a value close to 1 means that culture is highly likely to be negative. The Hosmer and Lemeshow test was used to assess how well the model fit with the data (the null hypothesis is that the model is an adequate fit). The 95% confidence interval for odds ratio was assessed for every predictor. Deviance was used to compare the logistic regression models (the higher the deviance, the less adequate the model is). The pROC package was used to plot receiver operating characteristic (ROC) and to calculate the area under the ROC curve (AUC). We assessed sensitivity, specificity and AUC (95% CI) for every model.

Also, optimal cut-off values of all hematological parameters were calculated by time-dependent receiver operating characteristic curves as prognostic factors for treatment outcome in TB. The optimal cut-off value for each biomarker was determined to maximize predictive specificity and sensitivity using the maximum value of Youden’s index [38], which maximizes sensitivity-(1 -specificity).

The AUC was used to assess prognostic accuracy for models and for every biomarker, which ranges from 0.5 to 1.0, 0.5 indicating no discriminative ability and 1.0 indicating highest detection accuracy. It is considered outstanding if AUC is bigger than 0.9, excellent if AUC is between 0.80 and 0.89, and acceptable if AUC is between 0.7 and 0.8.

## Results

In this study, the 106 newly diagnosed PTB patients were evaluated according to the major exclusion criteria, after which 90 (84,9%) patients above 18 years were selected. A number of 63 patients have negative culture conversion after 2 months of TB treatment.

**Fig 1:**
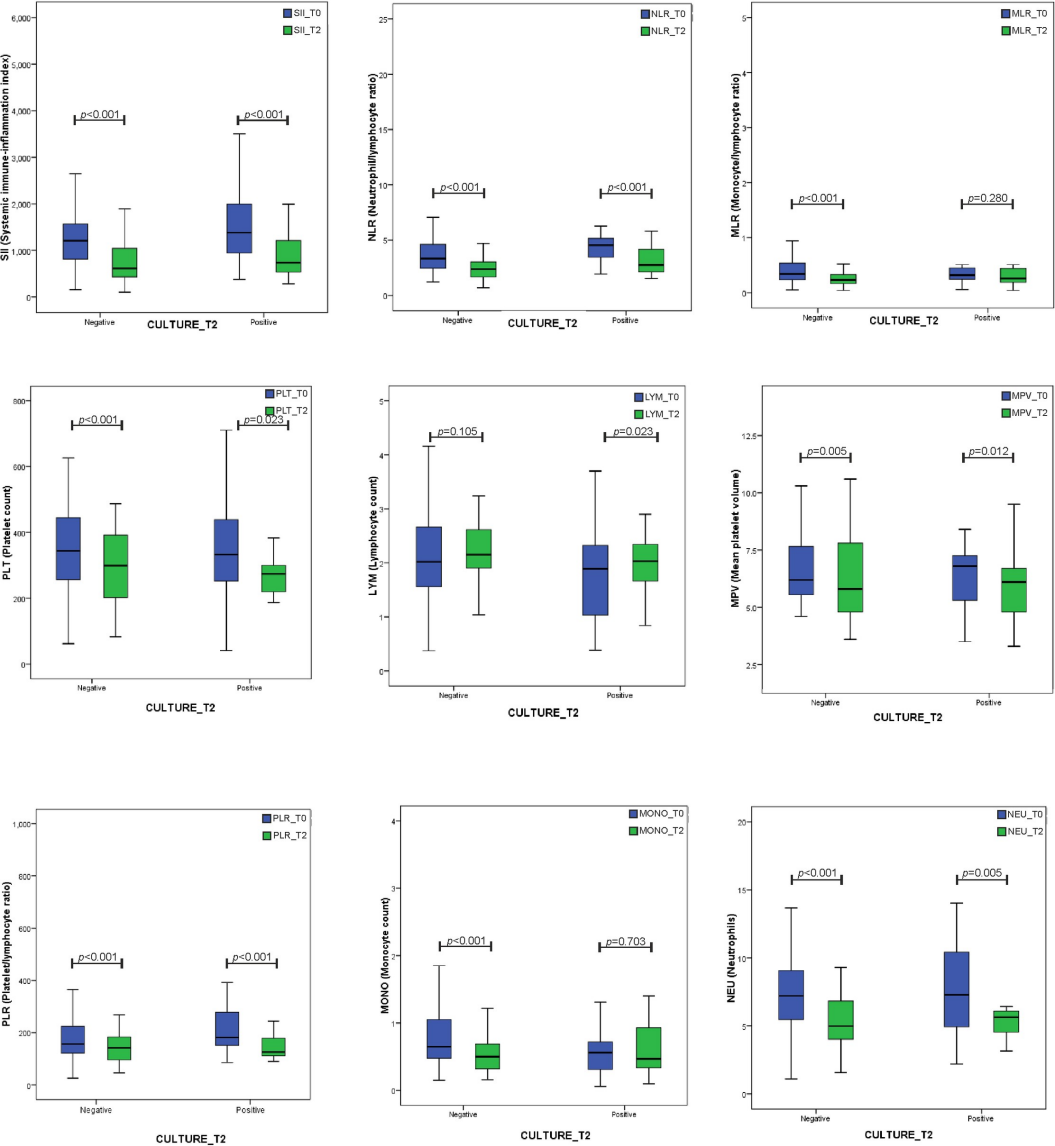
Comparative box-plots for some hematological parameters. Box-and-whisker plots representing median of SII, NLR, MLR, PLT, LYM, MPV, PLR, MONO and NEU levels. Blue box designates a boxplot of value at T0. Green box designates a boxplot of the value at T2. The horizontal line inside the box indicates the median value. Whiskers extend to the largest and smallest observed values within the box lengths. In each box plot, P_(Negative)_ = T0 vs. T2 for patients turned negative culture at T2 (in left), n = 63 and P_(Positive)_ = T0 vs. T2 for patients remained positive culture at T2 (in right), n = 27. Statistically significant deviations between groups (p < 0.05) are indicated by a horizontal line.

We assessed if the values of HCT, MCV, RDW-CV, RDW-SD, MCH, MCHC, WBC, LYM, NEU, MON, PLT, PCT, PDW-CV, PDW-SD, NLR, PLR, MLR, SII and ESR differ significantly when compared to the patients before treatment (T0) versus after the 2 month regimen treatment (T2). As shown in Fig 1 showing the comparative box plots for representative hematological parameters and **S2 Table** including the comparative analysis of the 23 hematological parameters, according to statistical analysis, it was observed that WBC, NEU, MONO, Hb, MCV, PLT, PCT, ESR, SII, NLR, MLR, LYM, MPV and P-LCR values were significantly lower in the 63 PTB patients that turned culture negative after treatment (T2) than in the same patients that were culture positive prior to the treatment (T0).

The MLR, NLR, PLR and SII values were also significantly lower when compared to the 63 patients that turned culture negative (T0 vs. T2) reaching the statistical significance (p<0.001). The lower MLR, NLR, PLR ratios and SII index were consistently associated with significantly lower absolute monocyte counts (Median: 0.5, IQR: 0.32–0.72), absolute neutrophils counts (Median: 4.97, IQR: 4–6.87) and absolute platelets counts, respectively (Median: 299, IQR: 202–394), but slightly non significantly higher absolute lymphocyte counts (Median: 2.15, IQR: 1.89–2.62).

When analyzing the hematological parameters between the 27 positive patients after treatment (T2) versus the same patients that were culture positive prior to the treatment (T0), significantly (p<0.001) elevated Hb, HCT, RDW-CV and RDW-SD values were observed, as shown in **S2 Table**. Among the median, there was a statistical difference between these groups in terms of NLR (Median: 2.75, IQR: 2.13–4.17) and PLR (Median: 125.5, IQR: 110.4–194.1) ratios, or SII index (Median: 739.6, IQR: 476.9–1215). Thus, the NLR, PLR and SII were all significantly lower in the 27 positive patients after treatment (T2) than in the same culture positive patients prior to the treatment (T0). These significantly lower values were associated with slightly significant higher absolute lymphocyte counts (Median: 2.03, IQR: 1.65–2.35, P = 0.023).

The comparative analysis between the two medical centers involved in this study (comprised in Table 1), concluded that the hematological parameters do not vary significantly by center.

**Table 1:** Comparison of the 23 hematological parameters by center.

Characteristics	Centre 1 (N = 49)	Centre 2 (N = 41)	p-value
Age	46.8±11.59	50.17±11.81	0.42
WBC_T0	11.29±3.55	9.85±3.6	0.4
LYM_T0	2.06±0.8	1.89±0.8	0.2
NEU_T0	8.08±3.19	7.03±3.3	0.4
MONO_T0	0.82±0.53	0.64±0.38	0.09
MLR_T0	0.48±0.63	0.37±0.22	0.4
NLR_T0	4.62±3.09	4.32±3.11	0.4
PLR_N0	220.86±159.73	200.78±125.1	0.67
SII_T0	1741.66±1257.43	1472.27±1264.07	0.4
RBC_T0	4.29±0.622	4.22±0.82	1
HGB_T0	119.24±22.38	121.51±25.0	0.85
HCT_T0	37.6±5.66	37.28±7.58	1
MCV_T0	87.97±7.48	88.79±9.72	0.85
MCH_T0	28.92±3.31	34.83±36.6	1
MCHC_T0	323.69±13.9	327.05±14.79	0.7
RDW_CV_T0	15.67±2.42	15.53±2.27	0.85
RDW_SD_T0	42.67±7.86	43.17±9.67	1
PLT_T0	380.12±145.55	326.29±143.83	0.2
PDW_CV_T0	39±1.1	39.31±1.27	0.42
PDW_SD_T0	19.26±3.3	17.59±2.39	0.034*
MPV_T0	6.93±1.78	6.36±1.33	0.18
PCT_T0	0.26±0.11	0.22±0.14	0.2
P_LCR_T0	25.27±6.34	25.51±4.85	0.85
ESR_T0	82.71±30.95	69.78±35.34	0.67
WBC_T2	9.29±3.23	8.09±2.37	0.2
LYM_T2	2.25±0.7	2.02±0.54	1
NEU_T2	6.05±2.89	5.18±2.03	0.28
MONO_T2	0.56±0.28	0.54±0.32	0.67
MLR_T2	0.27±0.15	0.29±0.2	1
NLR_T2	2.89±1.48	2.7±1.36	0.51
PLR_N2	151.59±77.81	155.72±80.06	1
SII_T2	972.27±747.68	869.36±695.28	0.2
RBC_T2	12.93±58.64	4.53±0.69	0.82
HGB_T2	129.82±14.86	127.15±15.57	0.4
HCT_T2	39.71±4.49	38.96±4.72	0.51
MCV_T2	87.85±7.33	85.07±12.81	0.82
MCH_T2	29.16±3.16	28.88±3.86	0.79
MCHC_T2	327.1±13.32	327.05±14.93	0.49
RDW_CV_T2	17.67±3.68	16.79±3.26	0.28
RDW_SD_T2	48.28±10.17	45.09±9.22	0.2
PLT_T2	307.41±104.61	289.76±103.17	0.38
PDW_CV_T2	39.86±2.07	38.91±3.7	0.97
PDW_SD_T2	18.27±3.1	16.76±1.68	0.09
MPV_T2	6.29±1.9	5.92±1.39	0.88
PCT_T2	0.18±0.07	0.16±0.05	0.51
P_LCR_T2	22.96±6.87	23.35±6.2	0.88
ESR_T2	60.22±37.42	60.49±39.53	0.94

Abbreviations: ESR: erythrocyte sedimentation rate; Hb: hemoglobin, HCT: hematocrit; RBC: red blood cell count; LYM: lymphocytes; MCV: mean corpuscular volume; MCH: mean corpuscular hemoglobin; MCHC: mean corpuscular hemoglobin concentration; MLR: monocyte-to-lymphocyte ratio; MON: monocytes; NEU: neutrophils; NLR: neutrophil-to-lymphocyte ratio; RDW-CV: red cell distribution width, coefficient variation; RDW-SD: red cell distribution width, standard deviation; PCT: plateletcrit; PDW-CV: platelet distribution width, coefficient variation; PDW-SD: platelet distribution width, standard deviation; PLR: platelet-to-lymphocyte ratio; PLT: platelets count; P-LCR: platelet large cell ratio; SII systemic immune-inflammation index and WBC: white blood cell count. Others: N: number of individuals; P-value: derived from Pearson chi-square

*p-value<0.05.

**Fig 2:**
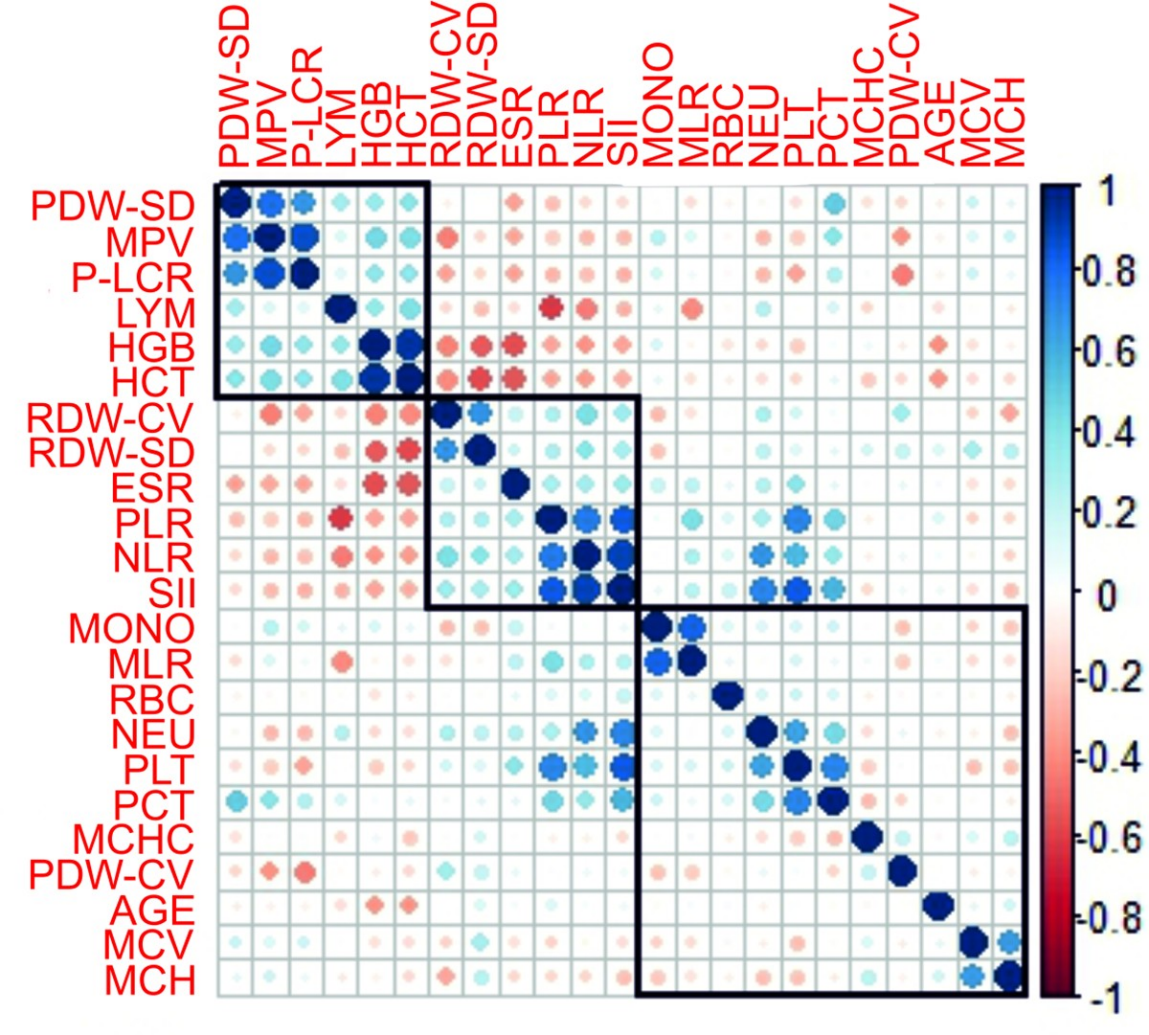
Correlogram with hierarchical clustering of covariates included in the logistic regression analysis. Positive and negative correlations are represented by blue and red dots. The sizes and the shades of the dots reflect the strengths of correlation between pairs of hematological parameters. Colours range from bright blue (strong positive correlation; i.e. r = 1.0) to bright red (strong negative correlation; i.e. r = −1.0). Correlations are ordered by hierarchical clustering with clusters outline. Figure drawn using the R corrplot package (https://github.com/taiyun/corrplot).

Spearman’s correlation analysis revealed significant positive correlations between NLR with NEU (r = 0.79, p<0.01), SII with NEU (r = 0.846, p<0.01), PLT with SII (r = 0.831, p<0.01), PLT with PCT (r = 0.71, p<0.01) and MPV with P-LCR (r = 0,897, p<0.01) in patients after 2 months of treatment, as shown in Fig 2.

The correlation values in the Spearman correlation matrix ordered by hierarchical clustering and visualized by correlogram identified three clusters of smaller groups of closely related hematological parameters, Fig 2.

These three obtained clusters containing smaller groups of closely related parameters helped us to analyze three more models by logistic regression, Models 11–13, Table 2.

**Table 2:** Logistic regression analysis of the models of predictors.

Model	Predictors	p-value from Hosmer and Lemeshow Test	Deviance	Sensitivity/ specificity/overall correct classification rate	AUC (95% CI)	p-value from predicted probability
Model1	Age, LYM, NEU, MLR, SII	0.370	100.315	90.5%/22.2%/70%	0.689 (0.577–0.801)	0.005
Model2	LYM, NEU, MLR, SII	0.008	101.064	90.5%/14.8%/67.8%	0.688 (0.577–0.799)	0.005
Model3	LYM, NEU, MLR, SII, RDW-CV	0.167	98.661	90.5%/18.5%/68.9%	0.705 (0.595–0.814)	0.002
Model4	LYM, NEU, MLR, SII, RDW-CV, HCT	0.111	97.856	90.5%/25.9%/71.1%	0.724 (0.615–0.833)	0.001
Model5	LYM, NEU, MLR, SII, RDW-CV, HCT, PCT	0.113	97.468	90.5%/14.8%/67.8%	0.731 (0.624–0.838)	0.001
Model6	Age, LYM, NEU, MLR, SII, RDW-CV, HCT, PCT	0.122	97.23	92.1%/25.9%/72.2%	0.735 (0.629–0.842)	<0.001
**Model7**	**Age, LYM, NEU, MLR, PLR, SII, RDW-CV, HCT, PCT**	**0.493**	**96.743**	**90.5%/29.6%/72.2%**	**0.735 (0.626–0.843)**	**<0.001**
Model8	Age, WBC, LYM, NEU, MLR, PLR, SII, RDW-CV, HCT, PCT	0.738	96.390	92.1%/22.2%/71.1%	0.728 (0.616–0.841)	0.001
Model9	Age, WBC, LYM, NEU, MLR, PLR, SII, RDW-CV, HCT, PCT, MCH	0.874	96.365	92.1%/22.2%/71.1%	0.725 (0.609–0.842)	0.001
Model10	Age, LYM, NEU, MLR, PLR, SII, RDW-CV, PCT	0.136	96.88	90.5%/25.9%/71.1%	0.728 (0.618–0.839)	0.001
Model 11	PDW_SD, MPV, P_LCR, LYM, HGB, HCT	0.135	100.932	92.1%/18.5%/70%	0.698 (0.580–0.817)	0.003
Model 12	RDW_CV, RDW_SD, ESR, PLR, NLR, SII	0.131	101.221	98.4%/18.5%/74.4%	0.660 (0.538–0.781)	0.017
Model 13	MONO, MLR, RBC, NEU, PLT, PCT, MCHC, PDW_CV, Age, MCV, MCH	0.213	95.307	92.1%/25.9%/72.2%	0.713 (0.598–0.829)	0.001

Abbreviations: HCT: hematocrit; LYM: lymphocytes; MCV: mean corpuscular volume; MCH: mean corpuscular hemoglobin; MLR: monocyte-to-lymphocyte ratio; MON: monocytes; NEU: neutrophils; NLR: neutrophil-to-lymphocyte ratio; RDW-CV: red cell distribution width, coefficient variation; PCT: plateletcrit; PLR: platelet-to-lymphocyte ratio; PLT: platelets count; SII systemic immune-inflammation index and WBC: white blood cell count.

The best model of predictors is shown in bold.

The highest positively correlated parameters presented in the correlogram were MPV with PDW-SD and PL-CLR with MPV (Spearman’s correlation coefficient ≥ 0.8).

As shown in Table 2, the logistic regression analysis revealed the best models for predictors.

**Fig 3:**
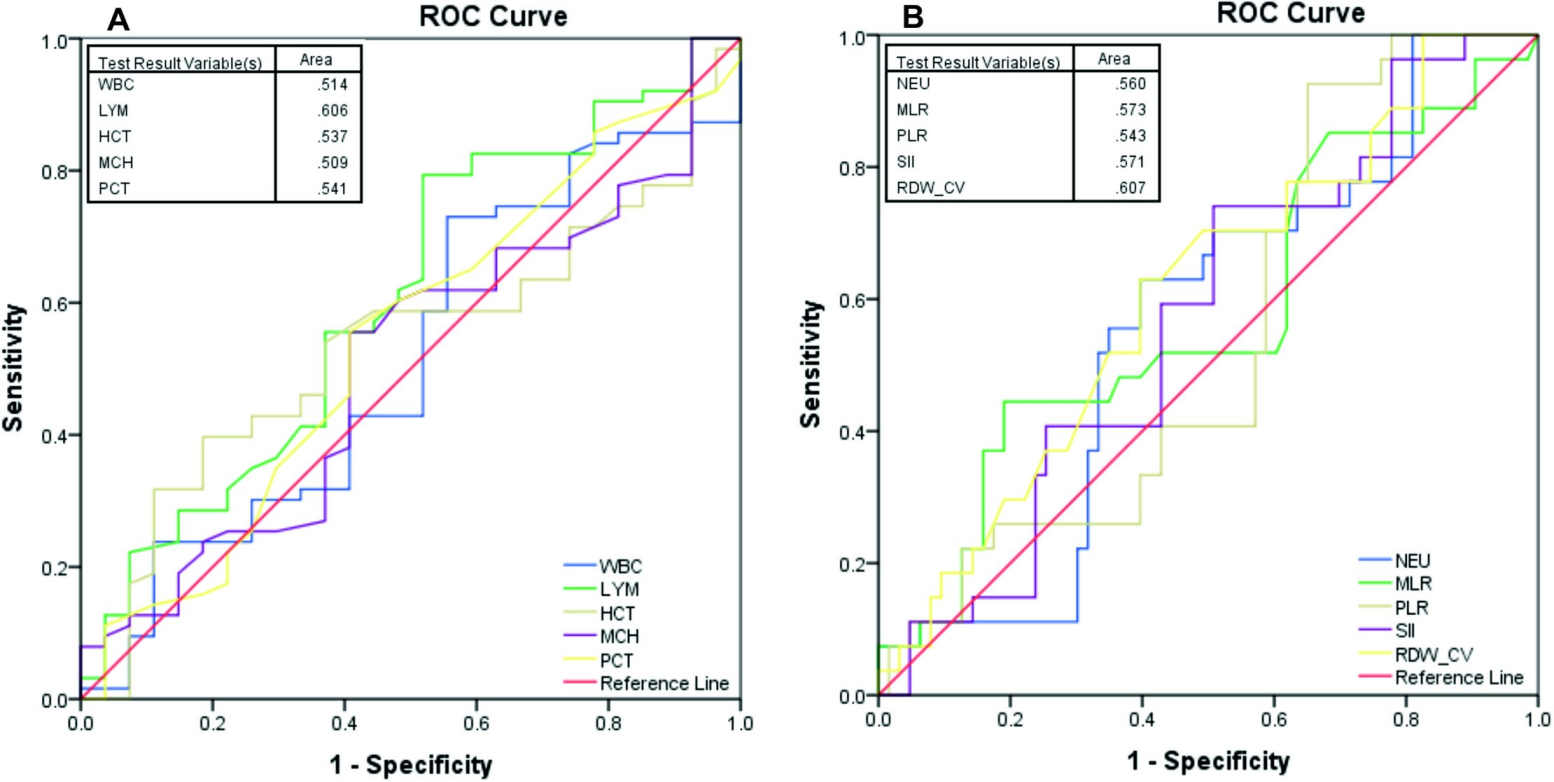
Receiver operating characteristic (ROC) curves for the representative single hematological parameters. (A) The analysis of AUCs (Area under the curve) for WBC, LYMF, HCT, MCH and PCT and (B) NEU, MLR, PLR, SII and RDW-CV indicated no statistically significant results, except LYM. Abbreviations: AUC: Area Under the Curve; ROC: Receiver Operating Characteristic; HCT: hematocrit; LYM: lymphocytes; MCH: mean corpuscular hemoglobin; MLR: monocyte-to-lymphocyte ratio; NEU: neutrophils; RDW-CV: red cell distribution width, coefficient variation; PCT: plateletcrit; PLR: platelet-to-lymphocyte ratio; SII systemic immune-inflammation index and WBC: white blood cell count.

ROC curve was used to evaluate the diagnostic value of single markers WBC, LYM, HCT, MCH, PCT, NEU, MLR, PLR SII and RDW-CV (Fig 3). In analysis of all AUC (area under the ROC curve), the single biomarkers revealed no statistically significant results, except LYM for CULTURE (where we could establish the best cut-off that maximizes sensitivity and specificity of 1.83). With AUC less than 0.7 out of a possible 1.0, the analyzed hematological parameters are not good indicators to anticipate CULTURE, after 2 months of treatment.

**Fig 4:**
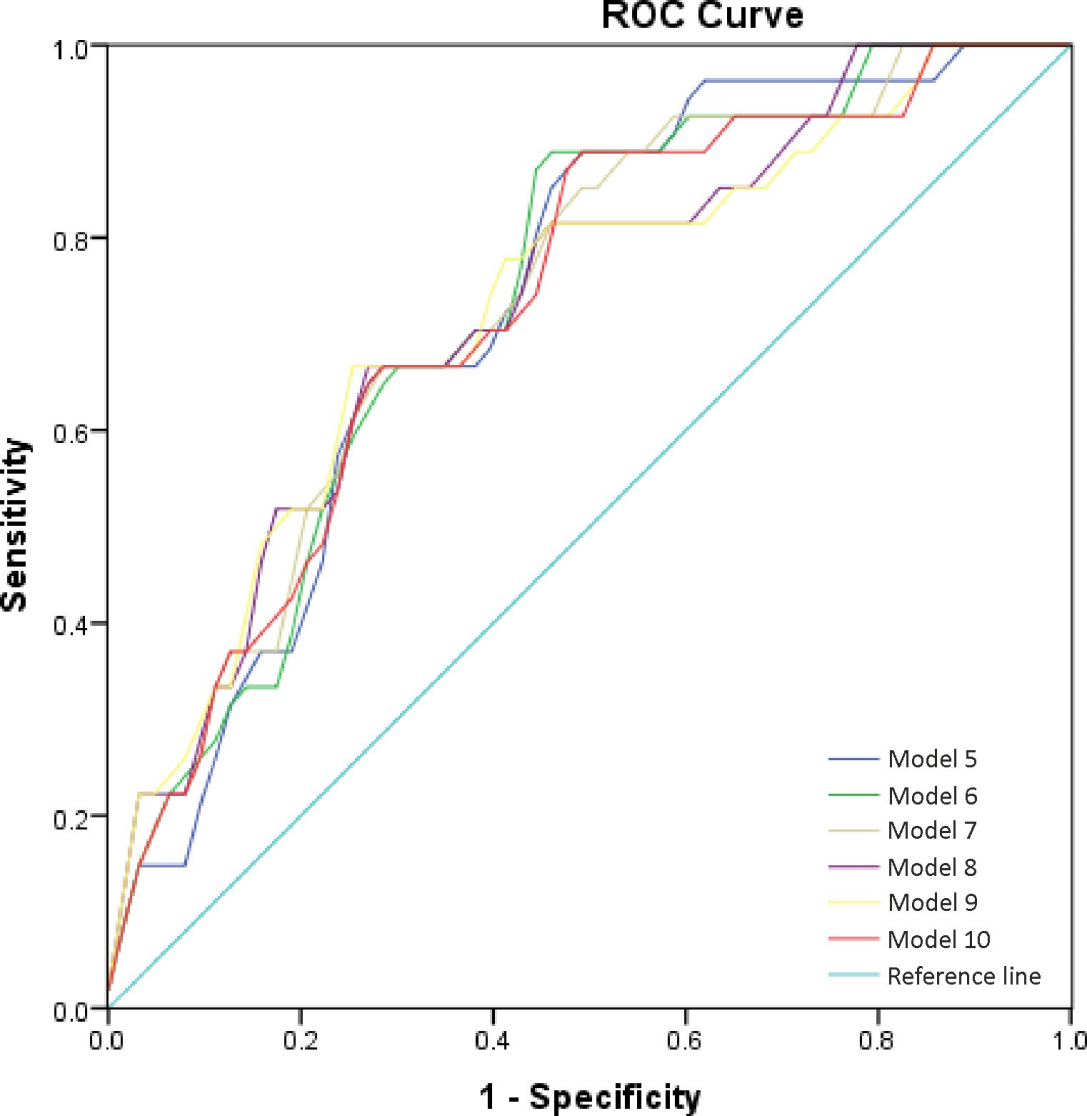
Receiver operating characteristics (ROC) curves for different models calculated from the logistic regression with best AUC. Abbreviations: AUC: Area Under the Curve; ROC: Receiver Operating Characteristic; The Models 11–13 as represented in Table 2.

Next, we sought to integrate our single hematological parameters into models with combined parameters. A combination of more than one discriminatory hematological parameter was developed via logistic regression analysis. In the ROC curve analysis, the results displayed that the AUCs for these models were significantly higher than the AUCs for the single hematological parameters. The value of the combined predictive models of multiple markers is indicated in Table 2. Accuracy of the models, evaluated by AUC as in Fig 4, suggested Model 7 (covariates Age, LYM, NEU, MLR, PLR, SII, RDW-CV, HCT, PCT) being the best from all proposed ones. The results indicated the best diagnostic value for CULTURE with an AUC of 0.735 (95% CI: 0.626–0.843).

## Discussion

Several studies have evaluated the importance of hematological parameters for predicting the risk of TB disease development, identifying the active tuberculosis infection or monitoring response to antimicrobial therapy [32, 39–41]. A considerable modification in the use of routine blood tests needs to be adequately applied for the diagnosis of active tuberculosis. In infectious or inflammatory diseases including pulmonary tuberculosis infection, complete blood count parameters and their ratios have been suggested as diagnostic markers. Some studies have proposed the use of these parameters and the ratios of various immune cell populations in peripheral blood as discriminating TB markers from non-TB infectious lung diseases [51].

There are limited studies which have shown that hematological parameters play an essential role in treatment strategies [42] based on the correlations between the levels of these markers in the peripheral blood with the outcomes of treatment against PTB.

To the best of our knowledge, this is the first study that has evaluated the correlation of this combination of hematological parameters and ratios with culture conversion after the first two months of treatment. Culture conversion at two months of anti TB therapy is of paramount importance in the evaluation of the effectiveness of antimicrobial therapy.

In our group restricted to patients with a definite diagnosis, we found that within the hematological parameters, values of WBC, NEU, MONO, Hb (hemoglobin), MCV, PLT, PCT, ESR and P-LCR were significantly reduced in culture negative patients after the two month treatment compared to the same patients that were culture positive prior to the treatment (T0).

Similar results with our findings about WBC have previously reported that anti-TB chemotherapy can reverse WBC count that is increased in PTB subjects due to the elevated count of polymorphonuclear leukocytes and macrophages during bacterial infection [40, 43, 44]. However, other studies have shown no statistically significant difference on WBC count before initiation and after the completion of intensive phase of treatment [45, 46]. An increase of the proportion of TB patients with low WBC count after completion of the 2-month tuberculosis treatment in patients with PTB has been observed [45]. Goodman et al. [47] suggested that an increased WBC count is not a sensitive parameter of bacterial infection when compared to NLR that is accordance with other studies suggesting that WBC had low diagnostic performance for infections [48].

As a cause of TB-associated anemia, the inflammatory mediators have been shown to suppress the erythropoiesis [48]. Interestingly, in our study hemoglobin value significantly decreased after the implementation of anti-TB treatment in the patients with negative culture conversion. Such effect may be due to the hematologic toxicity of anti-TB treatment considering that the drugs can induce a reduction of Hb concentration [45]. Conversely, other previous studies have shown a consistent increase in Hb concentration after the anti-TB therapy in PTB patients [49]. On the other hand, a recent study suggested that mycobacterial burden influences the hemoglobin concentration in more susceptible anemia individuals rather than in every TB patient [50].

An inverse correlation between Hb values and ESR in conditions associated with inflammation or infection has been reported [51]. In fact, a persistent inflammatory status is associated with sustained elevated levels of ESR and other inflammatory markers such as CRP (C-reactive protein), which could be an effective indicator of anti-TB treatment failure. In present study, in accordance with that expected in TB patients, within the evaluated inflammatory parameters, ESR levels significantly decreased in patients that have turned culture negative, even if there is previous evidence that a degree of inflammation may be persisted after sputum conversion [52].

Herein, a significantly elevated platelet count was detected in culture positive patients prior to the treatment (T0) than in the same 63 patients that have turned culture negative (T2). Platelets are associated with inflammatory reactions and immune response. Plateletcrit (PCT) and platelet distribution width (PDW) have been recognized as inflammatory response markers closely clinically related to PLT activation. The increase in PCT that is nonlinearly correlated to the platelet count was found to be more distinctive than other platelet indices, PDW or MPV, for tuberculosis and its value decreases to normal levels after treatment [44]. In our study, in line with these observations, MPV positively clustered with PDW-SD in hierarchical cluster analysis based on the Spearman correlation coefficients. The PCT indicates changes in PLT level and MPV, being considered an analog of hematocrit. Our results indicate similarly to the findings of Tozkoparan et al. [53] that the level of PCT improved significantly reverting to lower levels after anti-TB therapy in the 63 patients that turned culture negative.

Furthermore, there are reports that documented a significantly higher platelet count in the PTB than the control groups while others showed a significant decrement of platelet count which was explained by the early diagnosis of patients [43]. Notably, it seems that the anti-inflammatory effect of the platelets during TB infection is triggered both by inducing platelet-leukocyte crosstalk and the platelet induced shift to a more anti-inflammatory response [54]. Besides, it was suggested that reduction in platelet count after the completion of the intensive phase of treatment could possibly be due to the toxicity of anti-TB drugs and the need for continuous monitoring and evaluation of TB patients for adverse hematological abnormalities.

Monocytes are critical for host immunity against Mycobacterium tuberculosis infection that activates innate and adaptive immunity [55]. An increased number of reports emphasize the multifunctional roles of neutrophils in host defense that may come into play as exposure to the bacillus in the early stage of infection [56, 57]. Both monocyte and neutrophil counts increase in TB and decrease significantly on patient’s discharge [30]. As expected, we observed that the monocytes and neutrophils values after the treatments were significantly lower in the PTB patients that turned culture negative.

Recently, several combined indicators, such us neutrophil–lymphocyte ratio (NLR), monocyte–lymphocyte ratio (MLR) and platelet–lymphocyte ratio (PLR) have been used as potential indicators to reflect inflammation and clinical diagnosis and prognosis evaluation, including as biomarkers of PTB severity [26, 58–60]. In the present work, on treatment we found that all these ratios declined significantly, suggesting that as the bacterial burden declines, the levels revert to lower values in the patients that turned culture negative. This dependence could indicate the usefulness of evaluation of these ratios before and after therapy in predicting clinical outcome. Additionally, we observed that the lower values of MLR, NLR and PLR in PTB patients that turned culture negative were consistently correlated with lower absolute monocyte, neutrophil and platelet counts, and non-significantly higher absolute lymphocyte counts. These findings may reflect the effectiveness of anti-TB therapy in patients with culture conversion.

Our results are consistent with the previously reported data indicating that the relative increase of monocytes is associated with the relative decrease of lymphocytes in PTB patients after treatment [26], but partially consistent with other studies that reported lymphocytosis in active TB cases, considering the inverse correlation with our results after the anti-TB therapy [30].

Similarly, we found that the NLR and PLR values in the 27 patients that remained culture positive after treatment decreased significantly and were associated with slightly higher absolute lymphocyte counts. Hence, we could assume that there was a trend in which these ratios were moving towards normal values.

Among all variables assessed in the current study, the other predictor we investigated after the TB therapy was the SII index. To the best of our knowledge, there is no other study that assessed the role of the SII index in predicting clinical outcome in PTB patients either in intensive or continuous phases of the anti-TB treatment. Similarly with the MLR, NLR and PLR ratios, we observed a significantly decreased level of the SII index in patients turned culture negative relative to the patients that remained culture positive after the 2 months of intensive treatment. Previous reports have demonstrated that SII is a better indicator of inflammatory and immune responses and could be an effective prognostic indicator for various types of disease associated with systemic inflammatory response [61].

Here, it was confirmed by logistic regression in a model for predictors adjusted by age, that a set of covariates could be predictors for culture negativity. Thus, logistic regression analysis was associated with higher probability for M. tuberculosis culture negativity at day 60 of anti-TB treatment (T2) for this model of predictors. These results could suggest that monitoring of these predictors may be a good indicator of treatment outcome assessment, but may present relatively high sensibility and specificity. Within the evaluated parameters, values of WBC, NEU, MLR, PLR, SII, and PCT decreased significantly after anit-TB therapy as discussed above and matched those observed in other studies. Additionally, Spearman’s correlation analysis revealed significant positive correlations between some of these covariates in patients that turned culture negative, such as between NLR with NEU, SII with NEW or PLT with PCT. However, our ROC curve results have shown that the AUC values of the single hematological parameter were not statistically significant for culture conversion, except the lymphocytes. Compared to single hematological parameters, combined parameters presented a slightly better ability to predict for culture conversion with the AUCs of some models greater than 0.7, reflecting a better diagnostic value.

Our study has several limitations. Even if the study was conducted at two centers, we included a relatively small sample size group of PTB patients. Although our study focused on the treatment outcomes, the inclusion of control groups would have been beneficial. We could not retrieve from registers other confounding factors including sociodemographic clinical factors because in some patients’ cards the information was not complete. We were only able to follow up data until after completion of the 2-month treatment. Also, molecular drug susceptibility testing was not considered in this study. Using the month 2 culture as a predictive marker for treatment outcome could suffer few limitations such us contamination and variable turnaround times. Various combinations with other markers could better assess the prediction of treatment outcome including the biochemical markers. Further large cohort studies in a multicenter are needed to validate our results with repetitive measurements data. Registering the data in different times of anti-TB therapy would better reflect the role of these factors in predicting the effectiveness of treatment. Additional follow-up studies are needed for outcome assessment after six-months of treatment based on these hematological parameters.

Despite these limitations, as far as we know, this is likely to be the first study to report a model using a large combination of routine blood parameters for prediction of effectiveness of intensive phase of anti-TB treatment in PTB patients. In summary, our study found a set of hematological parameters that declined significantly as the disease was treated, while the screening of others pointed out a minimal or non-significant role in predicting clinical outcome. Here, we described changes at day 60 of antitubercular therapy in some of the hematological parameters and ratios that reverted as a response to TB treatment. These findings could be useful for identifying a combination of covariates capable of assessing the effectiveness of anti-TB treatment, considering the important various data previously reported. Further studies should be performed to assess the hematological profiles that follow a similar change towards the 2-month treatment to indicate the clearance of mycobacteria.

## Supporting information

S1 Table Presents the socio-demographic data. (DOCX)

S2 Table Comparison of 23 hematological parameters values before the initiation of anti-TB therapy and after completion of the intensive phase of treatment. (DOCX)

S1 File Complete blood count by center. (XLSX)
