# Supplementary material for: Evaluation of prognostic significance of hematological profiles after the intensive phase treatment in pulmonary tuberculosis patients from Romania
Source: PLoS One. 2021 Apr 1;16(4):e0249301. doi: 10.1371/journal.pone.0249301 (PMC8016233; doi:10.1371/journal.pone.0249301)
Supplement: S1 Table — (DOCX) [file pone.0249301.s001.docx]

| **Socio-demographic factors** | **Study population** | | |
| --- | --- | --- | --- |
|  | **N** | **%** | **N missing** |
| **Gender** |  |  |  |
| Male | 64 | 71.1 |  |
| Female | 26 | 28.9 |  |
| **Age** |  |  |  |
| Mean Age | 48 |  |  |
| Media Age | 50 |  |  |
| **Age group** |  |  |  |
| 18-30 | 6 | 6.6 |  |
| 31-50 | 44 | 48.9 |  |
| >50 | 40 | 44.5 |  |
| **School Level** |  |  | 7 |
| Primary School | 29 | 34.9 |  |
| High School | 47 | 56.6 |  |
| College or University | 7 | 8.4 |  |
| **Area of residence** |  |  |  |
| Urban | 22 | 24.4 |  |
| Rural | 68 | 75.6 |  |
| **Occupational status** |  |  | 9 |
| Workers | 50 | 61.7 |  |
| Jobless | 31 | 38.3 |  |
| **Alcoholism** |  |  |  |
| Yes | 6 | 6.6 |  |
| No | 84 | 93.4 |  |
| **Smoking** |  |  | 6 |
| Yes | 78 | 92.8 |  |
| No | 6 | 7.2 |  |
