# Supplementary material for: Evaluation of prognostic significance of hematological profiles after the intensive phase treatment in pulmonary tuberculosis patients from Romania
Source: PLoS One. 2021 Apr 1;16(4):e0249301. doi: 10.1371/journal.pone.0249301 (PMC8016233; doi:10.1371/journal.pone.0249301)
Supplement: S2 Table — (DOCX) [file pone.0249301.s002.docx]

**S2_Table.** Comparison of 23 hematological parameters values before the initiation of anti-TB therapy and after completion of the intensive phase of treatment.

| **Parameter** | **T0 (total)** | **T0** | | **T2** | | **P values** | |
| --- | --- | --- | --- | --- | --- | --- | --- |
| Mean (SD)  Median (IQR) | **Culture positive**  **(N= 90)** | **T0**  **turned culture negative at T2)** | **T0**  **(remained positive at T2)** | **Turned culture negative**  **(N = 63)** | **Remained positive**  **(N = 27)** | **P1** | **P2** |
| WBC  (x 10^9^/L) |  |  |  |  |  |  |  |
|  | 10. (3.69)  10.54 (8.27-12.82) | 10.87 (3.47)  10.86 (8.48-12.67) | 10.08 (3.98)  9.34 (7.20-13.06) | 8.70 (2.83)  8.37 (7.11-9.78) | 8.84 (3.18)  8.7 (6.56-9.8) | **<0.001** | **0.039** |
| LYM  (x 10^9^/L) |  |  |  |  |  |  |  |
|  | 1.95 (0.82) | 2.09 (.79) | 1.73 (0.79) | 2.22 (.63) | 1.98 (0.64) | 0.105 | **0.023** |
|  | 1.97 (1.45-2.57) | 2.02 (1.54-2.67) | 1.89 (0.94-2.35) | 2.15 (1.89-2.62) | 2.03 (1.65-2.35) |  |  |
| NEU  (x 10^9^/L) |  |  |  |  |  |  |  |
|  | 7.57 (3.31) | 7.65 (3.33) | 7.49 (3.17) | 5.56 (2.51) | 5.89 (2.69) | **<0.001** | **0.005** |
|  | 7.25 (5.27-9.32) | 7.21 (5.36-9.08) | 7.28 (4.9-10.61) | 4.97 (4-6.87) | 5.64 (4.32-6.09) |  |  |
| MONO  (x 10^9^/L) |  |  |  |  |  |  |  |
|  | 0.73 (0.47) | 0.80 (.5) | 0.59 (0.36) | 0.53 (0.27) | 0.59 (0.35) | **<0.001** | 0.703 |
|  | 0.64 (0.47-.89) | 0.65 (0.47-1.14) | 0.56 (0.21-0.77) | 0.5 (0.32-0.72) | 0.47 (0.33-0.93) |  |  |
| MLR |  |  |  |  |  |  |  |
|  | 0.43 (0.48) | 0.46 (0.57) | 0.36 (.19) | 0.26 (0.14) | 0.33 (0.23) | **<0.001** | 0.280 |
|  | 0.34 (0.24-0.49) | 0.34 (0.22-0.56) | 0.32 (0.24-0.45) | 0.23 (0.16-0.34) | 0.26 (0.19-0.44) |  |  |
| NLR |  |  |  |  |  |  |  |
|  | 4.63 (3.37) | 4.15 (2.43) | 5.26 (4.21) | 2.66 (1.38) | 3.17 (1.47) | **<0.001** | **<0.001** |
|  | 3.59 (2.87-5.08) | 3.31 (2.4-4.67) | 4.54 (3.31-5.27) | 2.38 (1.64-3.05) | 2.75 (2.13-4.17) |  |  |
| PLR |  |  |  |  |  |  |  |
|  | 212.91(144.37) | 203.27 (149.86) | 231.40 (131.91) | 148.70 (75.17) | 164.59 (85.99) | **<0.001** | **<0.001** |
|  | 173.55 (123.18-240.28) | 156.7 (120.8-225.9) | 182.2(142.3-281.7) | 142.1 (96.1-184) | 125.5(110.4-194.1) |  |  |
| SII |  |  |  |  |  |  |  |
|  | 1618.94 (1260.59) | 1587.1(1339.64) | 1693.24(1073.27) | 901.94 (749.87) | 980.08 (662.96) | **<0.001** | **<0.001** |
|  | 1260.4 (811.15-1992.75) | 1205.2(8101-1587.2) | 1383.3(834-1998) | 615.7 (426-1076) | 739.6 (476.9-1215) |  |  |
| RBC  (%) |  |  |  |  |  |  |  |
|  | 4.26 (0.71) | 4.27 (0.78) | 4.23 (0.56) | 11.07 (51.71) | 4.51 (0.67) | **<0.001** | **0.001** |
|  | 4.21 (3.81-4.76) | 4.18 (3.78-4.92) | 4.21 (3.88-4.61) | 4.5 (4.22-5.07) | 4.41 (4.09-4.97) |  |  |
| HGB  (g/L) |  |  |  |  |  |  |  |
|  | 120.49 (23.29) | 123.59 (22.40) | 112.56 (24.62) | 128.81 (15.51) | 128.11 (14.59) | **0.003** | **<0.001** |
|  | 123.0 (105.75-130.00) | 125 (106-125) | 119 (101-127) | 124 (118-142) | 129 (119-136) |  |  |
| HCT  (%) |  |  |  |  |  |  |  |
|  | 37.55 (6.55) | 37.96 (7.06) | 36.25 (5.19) | 39.56 (4.66) | 38.92 (4.45) | **0.013** | **<0.001** |
|  | 38.05 (33.35-40.85) | 38.8 (33.5-41.5) | 37.4 (32.4-39.5) | 39.4 (36-43.4) | 38.7 (36.7-40.4) |  |  |
| MCV  (fL) |  |  |  |  |  |  |  |
|  | 88.49 (8.53) | 89.45 (8.82) | 85.76 (7.34) | 86.43 (11.21) | 86.95 (7.65) | **0.019** | **0.310** |
|  | 87.7 (83.75-92.35) | 89 (83.8-94.1) | 86.1 (83.6-89.9) | 86.8 (82.6-92.3) | 88.9 (82.9-91.7) |  |  |
| MCH  (pg) |  |  |  |  |  |  |  |
|  | 31.56 (24.56) | 32.98 (29.54) | 28.43 (3.81) | 29.16 (3.56) | 28.74 (3.32) | **0.639** | **0.102** |
|  | 28.25 926.6-30.9) | 28.4 (26.6-31.1) | 28.1 (27-29.6) | 28.5 (27.2-30.6) | 28.2 (27.3-30.5) |  |  |
| MCHC  (g/L) |  |  |  |  |  |  |  |
|  | 324.90 (14.62) | 326.56 (14.48) | 322.11 (13.73) | 325.75 (14.22) | 330.19 (13.18) | 0.628 | **0.005** |
|  | 326.0 (314.0-335.25) | 326 (315-337) | 323 (312-330) | 328 (313-335) | 333 (320-337) |  |  |
| RDW-CV  (%) |  |  |  |  |  |  |  |
|  | 15.61 (2.32) | 15.46 (2.36) | 15.95 (2.31) | 16.86 (3.38) | 18.22 (3.67) | **<0.001** | **<0.001** |
|  | 15.6 (13.9-17.33) | 15.6 (13.8-17) | 15.7 (14.2-17.8) | 16 (14.7-19.4) | 17.5 (15.6-19.8) |  |  |
| RDW-SD  (fL) |  |  |  |  |  |  |  |
|  | 43.04 (8.64) | 43.74 (9.62) | 40.95 (5.67) | 46.22 (9.70) | 48.23 (10.15) | 0.074 | **<0.001** |
|  | 41.5 (38.38-46.67) | 41.8 (39.4-46.7) | 39.9 (36.5-44.8) | 42.4 (38.7-56.5) | 44 (41.3-53.8) |  |  |
| PLT  (x 10^9^/L) |  |  |  |  |  |  |  |
|  | 351.04 (148.65) | 355.63 (139.70) | 355.52 (163.96) | 301.76(104.99) | 293.78(102.55) | **<0.001** | **0.023** |
|  | 337.5 (253.5-441.75) | 344 (257-452) | 333 (243-439) | 299 (202-394) | 274 (212-300) |  |  |
| PDW-CV  (%) |  |  |  |  |  |  |  |
|  | 39.13 (1.18) | 39.06 (1.05) | 39.34 (1.47) | 39.16 (3.21) | 40.04 (2.16) | 0.365 | 0.241 |
|  | 39 (38.6-39.2) | 39 (28.6-39.2) | 39.1 (38.6-39.2) | 39.1 (38.4-39.2) | 39.1 (38.4-43) |  |  |
| PDW-SD  (fL) |  |  |  |  |  |  |  |
|  | 18.61 (3.14) | 18.57 (3.09) | 18.36 (2.92) | 17.73 (2.67) | 17.24 (2.62) | 0.077 | **0.026** |
|  | 17.75 (15.8-20.6) | 17.7 (16-20) | 18.2 (15.8-20.6) | 16.8 (15.8-18.6) | 17.6 (14.8-18.7) |  |  |
| MPV  (fL) |  |  |  |  |  |  |  |
|  | 6.71 (1.63) | 6.75 (1.65) | 6.49 (1.52) | 6.21 (1.73) | 5.94 (1.60) | **0.005** | **0.012** |
|  | 6.6 (5.45-7.5) | 6.2 (5.5-7.7) | 6.8 (5.3-7.3) | 5.8 (4.8-7.8) | 6.1 (4.7-6.7) |  |  |
| PCT  (%) |  |  |  |  |  |  |  |
|  | 0.24 (0.13) | 0.24 (0.14) | 0.24 (0.1) | 0.17 (0.06) | 0.17 (0.07) | **<0.001** | **0.001** |
|  | 0.21 (0.16-0.29) | 0.21 (0.16-0.28) | 0.18 (0.16-0.32) | 0.17 (0.13-0.2) | 0.14 (0.13-0.2) |  |  |
| P-LCR |  |  |  |  |  |  |  |
|  | 25.55 (5.74) | 25.06 (5.41) | 26.12 (6.32) | 22.97 (6.69) | 23.51 (6.29) | **0.005** | **0.006** |
|  | 24.25 (21.15-30.07) | 23.78 (21.07-29.11) | 25.59 (22.14-30.23) | 23.6 (16.8-27.48) | 23.76 (17.8-27.37) |  |  |
| ESR  (mm/h) |  |  |  |  |  |  |  |
|  | 76.57 (33.15) | 74.49 (35.36) | 82.26 (28.45) | 58.75 (38.93) | 64.07 (37.79) | **<0.001** | **0.024** |
|  | 82 (55-100) | 80 (60-100) | 85 (50-110) | 55 (25-100) | 70 (40-95) |  |  |

Abbreviations: ESR: erythrocyte sedimentation rate; Hb: hemoglobin, HCT: hematocrit; RBC: red blood cell count; LYM: lymphocytes; MCV: mean corpuscular volume; MCH: mean corpuscular hemoglobin; MCHC: mean corpuscular hemoglobin concentration; MLR: monocyte-to-lymphocyte ratio; MON: monocytes; NEU: neutrophils; NLR: neutrophil-to-lymphocyte ratio; RDW-CV: red cell distribution width, coefficient variation; RDW-SD: red cell distribution width, standard deviation; PCT: plateletcrit; PDW-CV: platelet distribution width, coefficient variation; PDW-SD: platelet distribution width, standard deviation; PLR: platelet-to-lymphocyte ratio; PLT: platelets count; P-LCR: platelet large cell ratio; SII systemic immune-inflammation index and WBC: white blood cell count.

Others: N: number of individuals; SD: mean (standard deviation) IQR: median (interquartile range);

P-value: derived from Pearson chi-square; Significant differences are shown in bold (p < 0.001).

P1 = T0 vs. T2 for patients turned negative culture at T2 (63 patients) and P2 = T0 vs. T2 for patients remained positive culture at T2 (27 patients).
